# Nuclear Argonaute Piwi Gene Mutation Affects rRNA by Inducing rRNA Fragment Accumulation, Antisense Expression, and Defective Processing in *Drosophila* Ovaries

**DOI:** 10.3390/ijms21031119

**Published:** 2020-02-07

**Authors:** Anastasia D. Stolyarenko

**Affiliations:** Institute of Molecular Genetics, Russian Academy of Sciences, 2 Kurchatov Sq., 123182 Moscow, Russia; stol@img.ras.ru

**Keywords:** Piwi, piRNA, rRNA fragments, rRNA processing, antisense rRNA, 2S rRNA, 5.8S rRNA, 5S rRNA, ovaries, *Drosophila melanogaster*

## Abstract

*Drosophila* key nuclear piRNA silencing pathway protein Piwi of the Argonaute family has been classically studied as a factor controlling transposable elements and fertility. Piwi has been shown to concentrate in the nucleolus for reasons largely unknown. Ribosomal RNA is the main component of the nucleolus. In this work the effect of a *piwi* mutation on rRNA is described. This work led to three important conclusions: A mutation in *piwi* induces antisense 5S rRNA expression, a processing defect of 2S rRNA orthologous to the 3′-end of eukaryotic 5.8S rRNA, and accumulation of fragments of all five rRNAs in *Drosophila* *melanogaster* ovaries. Hypotheses to explain these phenomena are proposed, possibly involving the interaction of the components of the piRNA pathway with the RNA surveillance machinery.

## 1. Introduction

Animal gonad cells are equipped with a specialized system for silencing transposable elements based on homologous sequence recognition by short RNAs—the piRNA (Piwi-interacting RNA) pathway [1]. In eukaryotes, a conserved short-RNA based mechanism, the endosiRNA (endogenous small interfering RNA) pathway, also exists to fulfill a similar role [1]. Silencing transposable elements—dispersed genomic repeats capable of multiplication in the genome—is an important cellular task. Their fraction ranges from a few percent to the majority of a eukaryotic genome [2], including approximately one fifth of the *Drosophila* genome [3,4]. Their expression entails transpositions [5,6], resulting in mutations, genomic rearrangements, genome destabilization [7] and, at the organismal level, in sterility [8]. 

The endosiRNA pathway (siRNA, RNA-interference, RNAi) functions both in gonads, as well as in somatic tissues of *Drosophila* [9,10]. EndosiRNA ~21 *n*. are processed from double-stranded RNA by a double-stranded-RNA-specific RNase III endonuclease Dicer2 and loaded into Ago2, a short RNA binding protein of the Argonaute subfamily of the Argonaute protein family [11]. The resulting complex has been shown to guide sequence-specific cleavage of homologous RNAs in the cytoplasm (post-transcriptional silencing), leading to their degradation by the 5′-3′ exonuclease Xrn1 and the 3′-5′ exonucleolytic and endonucleolytic exosome [12]. RNAi has been found to act in heterochromatin formation pointing to its role in the nucleus [13], where its functions have been well-studied in the fission yeast *Schizosaccharomyces pombe* in the silencing of pericentromeric repeats thought to be transposon remnants [14]. Their transcripts are considered to be turned over by both RNAi-dependent and RNAi-independent mechanisms. The RNAi-dependent mechanism involves processing of repeat transcripts into endosiRNAs followed by the endosiRNA-containing complex RITS recruiting histone methytransferase Clr4 mediating heterochromatin formation (transcriptional silencing). The RNAi-independent mechanism involves RNA surveillance—the exosome-targeting complex TRAMP and the exosome, which is the main RNA surveillance machinery of the cell regulating maturation and degradation of a huge plethora of RNA species [15,16]. Surveillance pathways monitor all aspects of RNA biogenesis, including removal of nonfunctional transcripts arising from pervasive transcription and transcripts with a wide range of processing defects, arising from the complexity of nuclear RNA processing [15]. Surprisingly, noncoding RNA surveillance is largely accomplished by only a handful of ubiquitous, mostly exonucleolytic RNA decay enzymes, primarily including the exosome and 5′-3′ exonucleases Xrn2/Xrn1 [15,17,18]. The same core surveillance system degrades transcripts generated by RNA polymerases I, II and III [15]. Modulation and specificity of exosome activity requires additional protein cofactors, exosome-targeting complexes. Recently endonuclease Dicer has been found to have surveillance functions with a role of surveilling tRNA molecules [17] and shown to extensively control unstable antisense, bidirectional, and intergenic noncoding RNAs in fission yeast [18].

**Figure 1:**
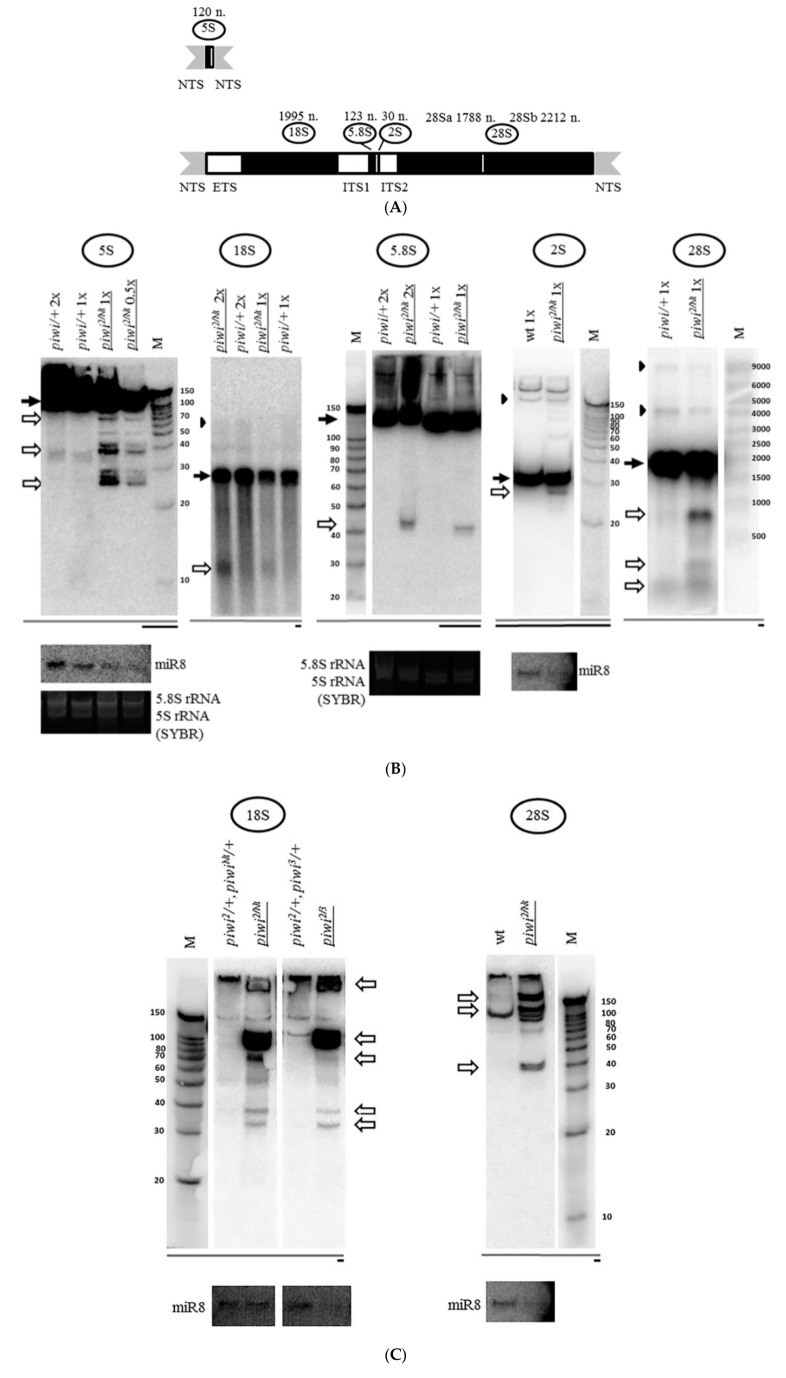

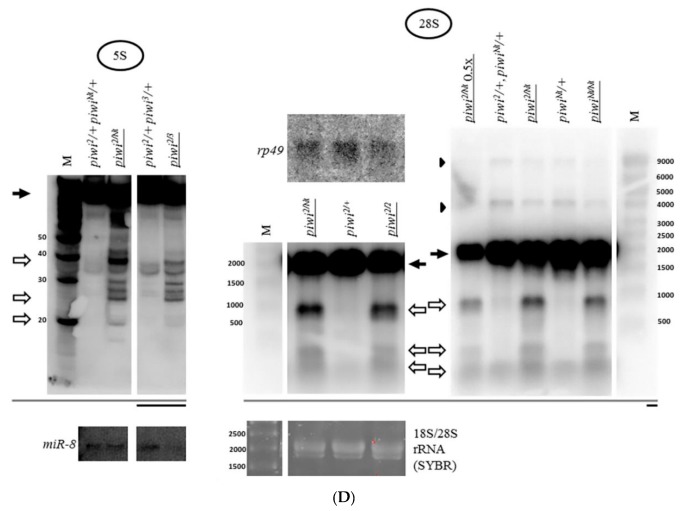
Mutation in the *piwi* gene causes the accumulation of fragments of all rRNAs in *Drosophila melanogaster* ovaries: (**A**) Schematic representation of a repeat of the tandem 5S rDNA cluster (top) and a repeat of the tandem 18S/28S rDNA cluster (bottom) in *Drosophila melanogaster*. The only ribosomal RNA polymerase III transcript, 5S rRNA, is produced as a precursor of 135 *n*. from the tandem 5S rDNA repeats. Nucleolar rRNAs are transcribed by RNA polymerase I as a single pre-rRNA precursor 8093 *n*. in size from the tandem 18S/28S rDNA repeats, processed to produce 18S, 5.8S, 2S, and 28S rRNA (divided into two parts—28Sa and 28Sb). Regions encoding mature rRNAs are shown in black and their sizes are indicated above. Transcribed spacers are shown in white: ETS: external transcribed spacer; ITS1, and ITS2: internal transcribed spacers 1 and 2. Nontranscribed spacers are shown in gray: NTS: nontranscribed intergenic spacer. (**B**) Transheterozygous combination *piwi^2/Nt^* leads to the accumulation of fragments of all rRNAs in *Drosophila melanogaster* ovaries. Shown are the results of Northern blot analysis of ovarian RNA with probes henceforth selected to detect the 3′-terminus of an rRNA species (except Figure 2 and Supplementary Figure S3). In the case of 2S rRNA the whole rRNA is detected, as its length is equal to the length of the probe (30 *n*.). The positions of the probes are henceforth depicted in coarse schemes below the Northern blot images as black bars with mature species shown as dark grey bars. The effect of accumulation of fragments (henceforth white arrows) is observed for all rRNAs. In most cases, the signals of mature rRNAs (henceforth black arrows) are brought to relative saturation, so that the fragments can be seen more clearly. rRNA precursors are marked with black arrowheads where apparent. The analyzed genotypes are indicated above the lanes (with mutant genotypes henceforth underlined): *piwi^2/Nt^* and control *piwi*/+, where *piwi*/+ is henceforth a mixture of heterozygous siblings *piwi^2^*/+ and *piwi^Nt^*/+. The wt genotype did not contain a mutation in *piwi* (*nos-GAL4* driver line). Equal amounts of total RNA were loaded per lanes to be compared, which is indicated by the symbols 2×, 1×, or 0.5× for relative RNA quantity. Rehybridization with a probe to detect *miR-8* microRNA or total RNA staining by SYBR Green II of the corresponding gels before blotting (labeled as SYBR) was used to control for loading. M henceforth indicates the lane with a marker, marker sizes in nucleotides are indicated. (**C**) For long 18S and 28S rRNAs, in addition to large fragments, smaller fragments are also detected in *piwi* mutants. For 18S (left) and 28S rRNA (right), fragments of the size range from short RNAs and above are observed. (**D**) The fact of rRNA fragment accumulation does not depend on a specific defect in the *piwi* gene. Fragments accumulate not only in transheterozygous *piwi^2/Nt^* and homozygous *piwi^Nt/Nt^* ovaries where Piwi has lost its nuclear localization (exemplified by 28S rRNA on the right), but also in the strong transheterozygous *piwi^2/3^* combination (exemplified by 5S rRNA on the left) and in null homozygous *piwi^2/2^* ovaries (exemplified by 28S rRNA in the middle). To all the lanes of each blot equal amounts of total RNA was loaded or a 0.5× amount where indicated. Rehybridization with probes to detect *miR-8* microRNA and ribosomal protein *rp49* mRNA or total RNA staining by SYBR Green II of the corresponding gels before blotting henceforth was used to control for loading. *Drosophila* long rRNAs have similar sizes of 1778 *n*. for 28Sa, 1995 *n*. for 18S, and 2212 *n*. for 28Sb, which frequently yields two signals in 1% agarose gels upon SYBR Green II staining.

In addition to controlling transposons, as well as exogenous threats like viruses [1], the RNAi pathway and its component proteins have several functions connected with rRNA. In eukaryotes, 18S, 5.8S and 28S rRNAs are encoded in a unit transcribed by RNA polymerase I as a single precursor molecule (Figure 1A) [19]. *Drosophila* also contains 2S rRNA which is orthologous to the 3’ end of 5.8S rRNA of other animals [20] (Figure 1A). *Drosophila* 28S rRNA is divided into two parts—28Sa and 28Sb [21]. There are usually over a hundred of tandemly-repeated 18S/28S rDNA units per haploid genome organized in a head-to-tail manner [19,22]. In *Drosophila*, the pre-rRNA precursor containing the rRNAs, 5′-external transcribed spacer (ETS) and two internal transcribed spacers (ITS1 and ITS2) (Figure 1A), have been shown to be processed by at least two alternative pathways to yield mature rRNAs of the small and large ribosomal subunits [19]. The processing is carried out mostly in the nucleolus (and nucleus) by multiple steps of endonucleolytic cleavage and exonucleolytic trimming reactions, yielding mature rRNAs in the cytoplasm. The nucleolus is the site of cytological manifestation of synthesis and processing of rRNA. Importantly, rRNA processing is carried out by machinery overlapping with that of RNA surveillance, including the exosome. 5S ribosomal RNA is encoded by a separate tandemly-repeated gene cluster transcribed by RNA polymerase III containing over a hundred units per haploid genome arranged in a head-to-tail manner [19,23]. These genes do not form a nucleolus-type structure, but the incorporation of 5S rRNA into the ribosomal subunits takes place in the nucleolus [23]. Among the functions of RNAi or its components connected with rRNA, RNAi is coordinated with heterochromatin in competing with RNA surveillance for substrates to appropriately partition potentially deleterious RNAs among these degradation pathways [24,25], studied in detail in fission yeast, including antisense RNA suppression at rDNA [26] and other loci. MicroRNA pathway (another small RNA pathway regulating various developmental programs and cellular processes) RNAse III Drosha, as well as Dicer and to a lesser extent Ago2 of the human microRNA/RNAi machinery [27], are required for the maturation of both ends of 5.8S rRNA [28]. Dicer, Ago-independently, in fission yeast protects the rDNA from replication stress resulting from transcription-replication conflicts involving antisense RNA polymerase II transcription at rDNA [29]. Finally, antisense transcription and small RNA response by RNAi are induced at rDNA by DNA double-stranded breaks [30] and can be required for their repair [31].

It should be noted that although representing distinct small RNA silencing pathways with distinct protein components, small RNA sources and sizes [32], piRNA and endosiRNA pathways have similarities and intersections. Animal piRNAs establish chromatin structure in way similar to endosiRNAs in fission yeast [33]. Moreover, it has been speculated that the competitive interaction of RNAi and exosome in yeast is conserved and replicated in the interaction of the piRNA pathway and the exosome in *Drosophila* in respect to transposable elements [34]. RNAi and piRNA have been found to interact with respect to different processes in different organisms, like transposable element silencing [35,36,37], antiviral functions [38,39], paramutation [40], and exogenous double-stranded RNA response [41,42,43]. RNAi and piRNA pathways can be differentially functioning in different organs: in *Drosophila* ovaries, Dcr2 and Ago2 are not required for fertility [44,45], in contrast with piRNA components [46], resembling mouse testes, while in mouse oocytes the situation is reverse [47]. 

As for the piRNA silencing pathway, although the generation of piRNAs is Dicer-independent [32], it can involve homologous sense and antisense RNA species with the potential to form a double strand [48]. The key components of the piRNA pathway are the conserved proteins of the Piwi subfamily of the Argonaute protein family [11], which bind short RNAs ~24–30 *n*. In the ovaries of *Drosophila*, the classical model for studying piRNA silencing, three such proteins are expressed, Piwi, Aubergine (Aub), and Argonaute3 (Ago3) [49], and comprise two parts of piRNA biogenesis, ping-pong and primary processing, working closely in the production of transposable-element derived short RNAs [50]. In case of defects in any of the core components, upregulation of transposable elements occurs [51]. 

The ping-pong amplification cycle involves cytoplasmic Aub and Ago3 proteins in the germline cells of the ovaries [49,52], multiplying in number the existing piRNAs from both the sense transcripts of the transposons themselves and transcripts of piRNA clusters that can be transcribed in antisense orientation with respect to transposons. This achieves the elimination of transposon transcripts at the post-transcriptional level in the cytoplasm. piRNA clusters are genomic collections of transposon fragments that serve as hotspots of piRNA production, involving both genomic strands (dual-strand clusters) and, more rarely, one strand (unistrand clusters), yielding long noncoding transcripts destined for processing [53]. Other proteins have been found to function in this process, including putative RNA helicase Spn-E capable of regulating the levels of Aub and Ago3 proteins [35].

The prevalently nuclear Piwi protein [54] in the germline cells of the ovaries is involved in producing primary piRNAs by phased processing of transcripts [55,56], extending the production of new piRNAs to more distant areas along the piRNA cluster transcript or transposon transcript from sites of piRNAs associated with Aub or Ago3. The phased processing is carried out by a specific piRNA pathway endonuclease Zucchini (Zuc). In somatic cells of the ovaries lacking Aub and Ago3, the primers for phasing are unclear, however, as a result of this process, piRNAs are also produced [57] and complexed with Piwi. The complex of piRNA and Piwi is transported to the nucleus [58] where it recruits histone lysine methyltransferase Eggless/SetDB1 installing heterochromatin marks recognized by heterochromatin protein HP1a and achieving the silencing of transposons at the transcriptional level [59]. Loading of Piwi with piRNAs ensured by the primary piRNA processing machinery, including RNA helicase Armitage (Armi), is necessary for Piwi nuclear localization and function [58,59,60] and their mutations result in dramatic cytoplasmic translocation of Piwi. 

Ping-pong has been proposed to compete with primary biogenesis for ping-pong-generated cleavage products [61]. Zuc endonuclease consumes piRNA precursors reducing their participation in ping-pong, and an Argonaute-only pathway can generate piRNAs in the absence of Zuc [61]. The ping-ping pathway is hypothesized to be an ancient small RNA-generating unit to which special endo- and exonucleases, like Zuc, were added in the course evolution [61].

Other functions of the piRNA pathway outside transposable element silencing are less studied. The primary processing pathway genes are known to be united by the fact that they are involved in the production of short RNAs from 3′ regions of mRNAs of a number of cellular protein-coding genes in *Drosophila* ovaries, as well as *Xenopus* eggs and mouse testes [62]. This shows that piRNA silencing subsystems and proteins can have special functions.

Not much is known about piRNA pathway involvement in the life of rRNA. In *Tetrahymena*, protein Twi12 of the Piwi subfamily of the Argonaute family was shown to interact with Xrn2 [63], a 5’–3’ exonuclease involved in the maturation of rRNA, RNA surveillance and termination [64]. Both of the knockdowns lead to defective 5.8S rRNA 5′-end maturation [63]. The lack of Twi12 loading with short RNAs prevents Xrn2 from entering the nucleus as part of a common complex [63], whereas loaded Twi12 appears to stimulate Xrn2 activity [65]. Xrn2 also functions in transcription termination characteristic of piRNA clusters, as shown in *Drosophila*, where it is normally inhibited by the Cuff protein in order to suppress premature transcription termination of the long piRNA precursor transcripts [66]. Additionally, piRNAs from 5S rRNA have been shown in mice to be coimmunoprecipitated with cytoplasmic Piwi subfamily protein Mili upon Tdrd1 mutation resulting in nuclear Piwi subfamily protein Miwi2 absence from the nucleus [67].

Regarding the connection of the Piwi protein and rRNA in *Drosophila*, it was shown that within the nuclei of ovarian cells the Piwi protein is distributed throughout the nucleoplasm, but is especially concentrated in the nucleolus [68,69]. In accordance with its classic function, Piwi has been demonstrated to repress R1 and R2 retrotransposons [68] that frequently insert into 28S rDNA in insects [70]. It was also shown that the Piwi protein determines the recruitment of the RNA polymerase I transcription factor Udd, a component of the SL1-like complex [71], into the nucleolus in the ovarian germline [72] with yet unclear functional consequences. The nucleolus is known to be enlarged and disintegrated in *Drosophila* in a number of piRNA silencing, chromatin and RNAi mutants in somatic cells and bulk *Drosophila* larvae [73]. For ubiquitous heterochromatic histone H3K9 lysine methyltransferase Su(var)3–9 and for Dicer2 mutants, this nucleolar disruption has been shown to be accompanied by a decrease in heterochromatic marks in the tandem rDNA locus and an increase in the number of ribosomal extrachromosomal circular DNA (eccDNA), demonstrating decreased stability of the genome in the locus [73], the latter shown for heterochromatin protein HP1a mutants as well [74]. However, the significance of the observations in respect to piRNA components remains to be clarified, as these effects are observed in the soma where the piRNA pathway is usually inactive [75]. The functions of Piwi in rRNA metabolism in the gonads where the piRNA pathway is active remain largely unclear.

In this work, it is shown for the first time that the *piwi* mutation and other mutations of the components of the primary piRNA processing pathway that dramatically affect Piwi localization in the nucleus, lead to a significant accumulation of fragments of all rRNAs in *Drosophila* ovaries. The fragments are usually shorter than mature rRNAs and may be short RNA-like in size. The processing of rRNA appears to be affected in terms of the maturation of 2S rRNA, the ortholog of the 3’ end of eukaryotic 5.8S rRNA. These phenomena are accompanied by the accumulation of antisense rRNA, demonstrated for 5S rRNA. Taken together, the obtained and literature data allow to suspect that Piwi could be involved in the proper metabolism of tandem rRNA repeats in the cells of the *Drosophila* ovary in a way which is different from its canonical function in silencing dispersed transposon repeats, including suppressing spurious piRNA generation at tandemly-repeated genes via nuclear Argonaute proteins, as well as a role of the piRNA pathway in safeguarding tandem repeat stability in the gonads.

## 2. Results

### 2.1. Mutation in the Gene Coding for piRNA Silencing Pathway Protein Piwi Causes Accumulation of Fragments of All rRNAs in Drosophila melanogaster Ovaries

The previously observed concentration of the key protein of the piRNA silencing pathway Piwi in the nucleolus of ovarian cells [68,69] led to the investigation of the effect of the mutation of the corresponding gene on ribosomal RNA. For this purpose, Northern analysis was carried out. Surprisingly, the transheterozygous *piwi^2/Nt^* combination leading to loss of Piwi from the nucleus by removal of its nuclear localization signal [51] caused accumulation of rRNA fragments, detected with probes to the 3’ ends of rRNAs (Figure 1B). This effect is observed both for the nucleolar transcripts of *Drosophila* RNA polymerase I (18S, 5.8S, 2S and 28S rRNA) and for the ribosomal transcript of RNA polymerase III (5S rRNA) (Figure 1A). In the mutants, the fraction of each individual fragment does not appear to be large in comparison with signals from the mature rRNA transcripts. However, it is much higher than that in heterozygous control ovaries, which can also demonstrate a set of weakly detectable fragments, overlapping in sizes with fragments in the mutants (Figure 1B). In these experiments, equal amounts of total RNA from mutant and control ovaries were used in all cases, unless otherwise indicated. When normalized to the total amount of RNA, the effects on the mature rRNA levels are not readily quantified, perhaps because the sum of rRNA fragments makes a contribution to the total rRNA that can appear as a decrease in the mature rRNA or pre-rRNA, because rRNA constitutes the predominant RNA component of cells. However, the accumulation of fragments remains the phenomenon that indisputably unites the response of different rRNAs to the mutation.

Loss of Piwi protein due to the null *piwi^2/2^* mutation [51,76] and the strong transheterozygous *piwi^2/3^* combination [76], as well as its removal from the nucleus upon *piwi^Nt/Nt^* mutation [51] lead to a similar effect of fragment accumulation (Figure 1D). This suggests that the fact of fragment accumulation does not depend on a specific defect in the *piwi* gene and on ovarian morphology, which is close to wild type in *piwi^2/Nt^* [51] and dramatically disturbed in *piwi^2/2^* [76].

Examining in detail the sizes of rRNA fragments accumulating in *piwi^2/Nt^* mutant ovaries, for the small-sized 2S rRNA 30 *n*. In length with a probe against the whole rRNA, an intense dominant fragment of approximately 27 *n*. is observed (Figure 1B, Figure 5), the length of which is characteristic of the piRNA silencing pathway, i.e., can be accumulated as a result of action of the intact Argonaute proteins of the Piwi subfamily. This analysis of fragment sizes is limited by the fact that the fragments can contain undetected polyA or polyU tails added in a nontemplated way, the possibilities of which cannot be excluded and require further analysis. Additionally, in order to be called a piRNA or endosiRNA, a fragment of a small RNA size needs to at least be coimmunoprecipitated with an Argonaute protein. Bona fide piRNAs and endosiRNAs homologous to rRNA have been described in Argonaute precipitates from *Schizosaccharomyces pombe* [26], *Neurospora crassa* [30], *Caenorhabditis elegans* [77], *Mus musculus* [67], *Drosophila melanogaster* S2 cells [78] and ovaries [79].

When analyzing fragments from the 3′-end of other mature rRNAs, it is obvious that the sizes of dominant fragments are not the same for different RNAs (Figure 1B) which may indicate various mechanisms of their formation, the possibilities of which arise when *piwi* is mutated. Another small sized rRNA, the 120 *n*. 5S rRNA, yields major fragments of lengths characteristic of the piRNA silencing pathway (~27, 28, 29 *n*.), similar to the size of the major 2S rRNA fragment, as well as ~38 *n*., however, both lower and higher molecular weight fragments can also be detected (Figure 1B). The formation of piRNA-sized fragments can be consistent with the previously published report of the loss of selectivity of the piRNA silencing pathway, when normal cellular transcripts, including the 5S transcript, are processed into piRNAs in piRNA pathway component Tdrd1 mutants [67].

**Figure 2:**
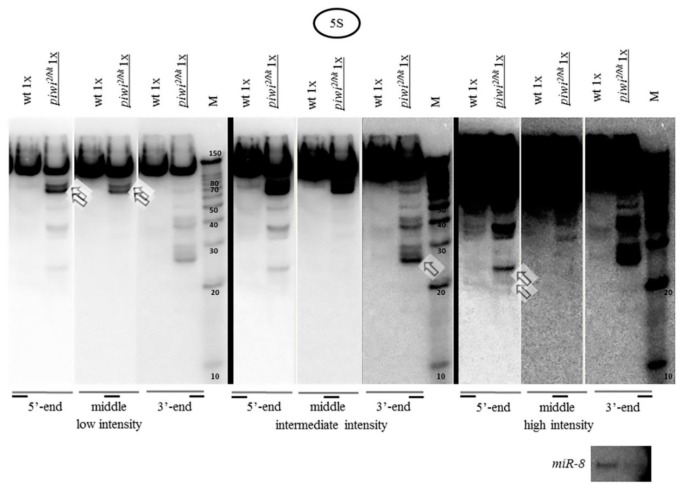
Fragments from different regions of 5S rRNA accumulated in *piwi* mutants are different in size: Hybridization of one blot with each of three probes detecting the 5′-terminus, middle and 3′-terminus of the 120 *n*. 5S rRNA was carried out by sequential reprobing and the results are presented in three different signal intensities increasing from left to right, separated by thick black lines. In the beginning of the molecule prominent fragments of ~70 and 80 *n*., as well as weak fragments of ~19, 21, 24 *n*., are observed, marked by squared white arrows. In the middle of the molecule major ~70 and 80 *n*. fragments are marked. In the end of the molecule prominent ~27, 28, and 29 *n*. fragments are marked. The wt genotype did not contain a mutation in *piwi* (*nos-GAL4* driver line).

Fragments from different regions of 5S rRNA preferentially accumulating in *piwi* mutants are different in size. Longer fragments (~70 and 80 *n*.) are detected as predominant in the beginning of the 5S rRNA molecule, as well as in the middle (Figure 2), which at first glance cannot be explained by the presumptive action of the intact Argonaute proteins or RNAi. This result suggests that the process of rRNA fragment accumulation is uneven and asymmetric with the middle of the molecule seeming to accumulate the least number of short fragments, being more protected from fragment accumulation. However, from the 5’ end, in addition, weak endosiRNA-sized fragments of ~21 *n*. can be observed, which could indicate the potential presence of double-stranded RNA processed by Dicer. This resembles the data in yeast [26,80], where in case of RNA surveillance defects, the RNAi pathway can gain access to abundant cellular transcripts. In addition, defects in RNA surveillance could also explain the accumulation of some rRNA fragments of sizes other than those of piRNAs or endosiRNAs.

For the 123 *n*. 5.8S rRNA the predominant fragment from the 3′-end was a somewhat longer fragment than for other rRNAs listed above of approximately ~42 *n*. (Supplementary Figure S3, right). A longer probe to the middle of the molecule (42 *n*. in length, presumably overlapping with this fragment by 13 *n*.) reveals the same fragment together with a longer ~81 *n*. fragment of comparable intensity (presumably overlapping with this fragment by the rest 29 *n*.) (Supplementary Figure S3, left). A similar fragment of ~82 *n*. from the 5’ end of 5.8S rRNA is also found normally in zebrafish somatic tissues and eggs [81,82]. Surprisingly, the sum of the lengths of these two fragments equals the length of mature 5.8S rRNA. Perhaps, 5.8S rRNA can be fragmented into two parts, which has already been proposed, assuming that the rRNA fragments were generated by a specific endonucleolytic cleavage process, rather than exonucleolytic digestion [81]. The analysis of the sequence and secondary structure [20] may allow to deduce the site of potential endonucleolytic cleavage. This site is likely located in the first loop or in the stem in close proximity to the first loop from the 3′-end of the *Drosophila* 5.8S rRNA molecule between A and C in the CACA context. Interestingly, the CA dinucleotide defines the poly(A) site for most protein-coding genes. Pre-mRNA is usually cleaved after the CA dinucleotide by CPSF endonuclease [83]. In order for processing to occur correctly, pre-mRNAs need specific cis-elements to guide the protein factors, including but not limited to, a U/GU-rich element located 10–30 *n*. downstream of the cleavage site [84]. This sequence feature is indeed present in the correct position in the mature *Drosophila* 5.8S rRNA. Thus, it is possible to assume that in the absence of Piwi 5.8S rRNA can become exposed to degradation in ovaries.

As for the large rRNA molecules, accumulation of major piRNA- or endosiRNA-sized fragments in their 3’ termini also does not appear to be readily observed (Figure 1C). For 18S rRNA 1995 *n*. long and 28S rRNA 3969 *n*. long, in its mature form divided into two comparable 28Sa and 28Sb portions in *Drosophila* (Figure 1A), major fragments with sizes of ~40 *n*., ~100 *n*. and over (Figure 1C) accumulate, as well as predominant discrete longer fragments of several hundred nucleotides: ~1200, ~900, and ~450 *n*. for 18S rRNA and ~900, ~400, and ~300 *n*. for 28Sb rRNA (Figure 1B). 

Thus, the disruption of the piRNA silencing pathway by mutations in *piwi* causes accumulation of fragments of various sizes of all five *Drosophila* rRNAs. The sizes of rRNA fragments in *piwi* mutants can indicate the participation in their accumulation of different small RNA pathways and RNA surveillance/degradation pathways.

### 2.2. Mutations in Other Primary piRNA Pathway Components, but not Ping-Pong Components, Cause a Significant Accumulation of rRNA Fragments

**Figure 3:**
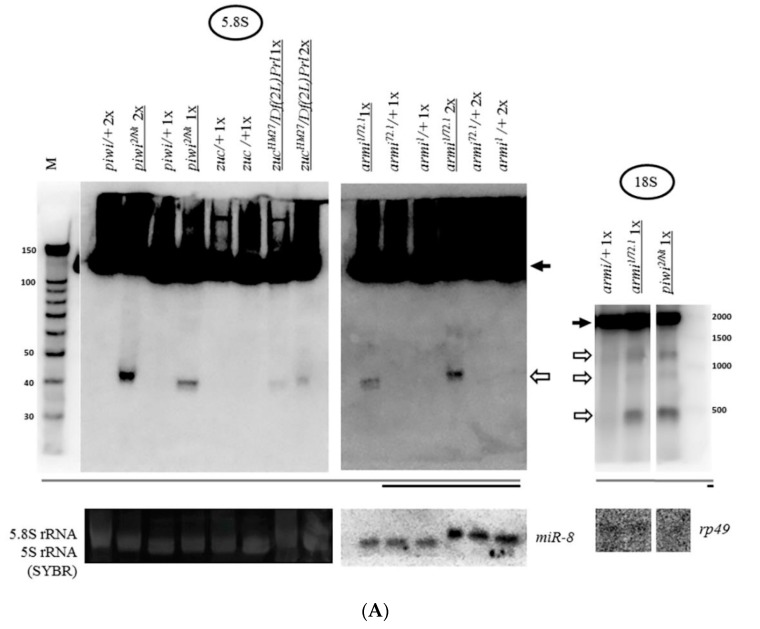

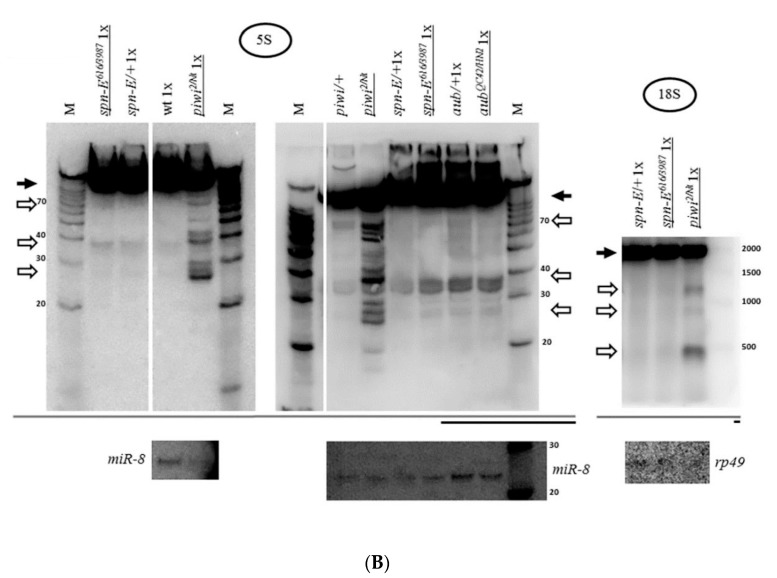
Mutations in other primary piRNA processing components, but not ping-pong components of the piRNA pathway, cause a significant accumulation of rRNAs fragments: (**A**) Effects of mutations in *zuc* and *armi* of primary piRNA processing. The fragments significantly accumulate in the transheterozygous *zuc^HM27^/Df(2L)Prl* ovaries (exemplified by 5.8S rRNA on the left) and *armi^1/72.1^* ovaries (exemplified by 5.8S rRNA in the middle and 18S rRNA on the right). Fragments are designated by white arrows, mature rRNAs by black arrows; (**B**) Effects of mutations in *aub* and *spn-E* of ping-pong amplification. The fragments do not significantly accumulate in transheterozygous *spn-E^616/3987^* ovaries (exemplified by 5S rRNA on the left and in the middle and 18S rRNA on the right) and *aub^QC42/HN2^* ovaries (exemplified by 5S rRNA in the middle). Equal amounts of RNA were loaded on lanes of controls and mutants to be compared in each blot.

Expansion of the study of the piRNA silencing pathway disruption effects on rRNA fragments revealed the same effect of fragment accumulation caused by mutations in other primary piRNA genes [60,85]—*armi^1/72.1^* [86] and *zuc^HM27^/Df(2L)Prl* [87] (Figure 3A). These mutations are united by the fact that they dramatically affect the localization of Piwi in the nuclei, without affecting ping-pong [88,89]. The frequently used mutation combinations disrupting the components of ping-pong amplification, *spn-E^616/3987^* [90] and *aub^QC42/HN2^* [91], do not lead to a significant accumulation of rRNA fragments (Figure 3B), while reported to largely maintain the characteristic localization of Piwi in the nuclei [88]. Importantly, in line with the canonical function of the piRNA pathway, these mutations comparably derepress transposable elements, as illustrated by model transposon *HeT-A* upregulation detected by RT-PCR (Supplementary Figure S1).

Thus, the presence of Piwi in the nucleus ensured by proper primary piRNA biogenesis is necessary to prevent the accumulation of rRNA fragments in *Drosophila* ovaries. It is likely that this necessity could manifest itself in all cell types where Piwi is expressed in the ovary. As part of the presented data, in the germline cells of the ovary, the need for Piwi in the nucleus can be illustrated by the effect of the transheterozygous combination *armi^1/72.1^*, which was shown to selectively disrupt Piwi localization in nuclei of nurse cells of germline nature [60,92] (Figure 3A). In somatic cells of the ovary, the transheterozygous combination *zuc^HM27^/Df(2L)Prl*, which appears to affect the localization of Piwi selectively in follicle cells of somatic nature [51], also leads to fragment accumulation (Figure 3A). Indeed, the effect of this combination appears to be somewhat weaker than that of the *piwi^2/Nt^* transheterozygote, which affects both germline and somatic cells.

**Figure 4:**
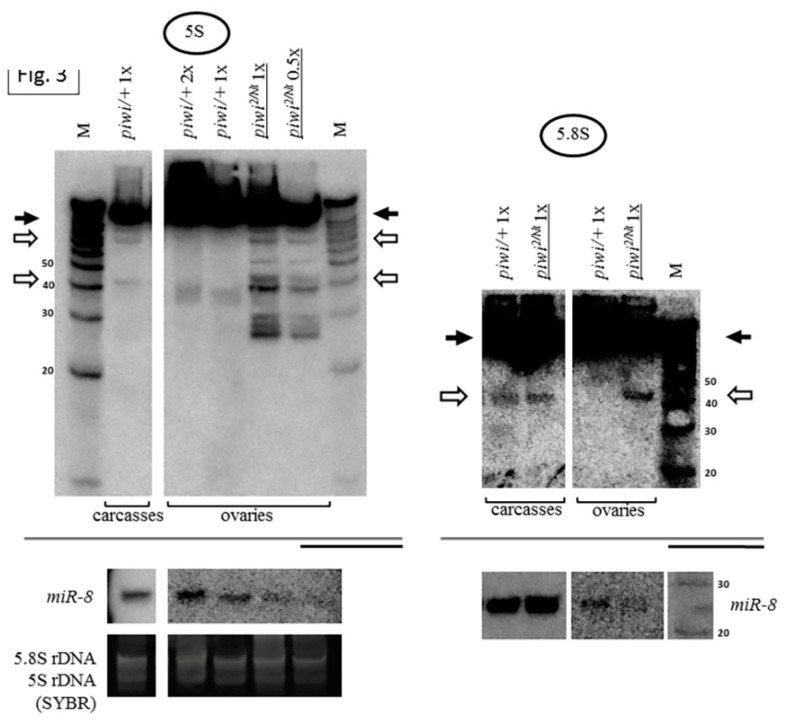
Carcasses (bodies without ovaries) of flies contain a set of rRNA fragments overlapping with that in *piwi* mutant ovaries: A number of fragments are present not only in the transheterozygous *piwi^2/Nt^* ovaries (which are several lanes from the blot shown in Figure 1B), but also in control carcasses (exemplified by 5S rRNA on the left and 5.8S rRNA on the right). Fragments are designated by white arrows, mature rRNAs by black arrows. The same amount of RNA from the ovaries and carcasses was loaded on the lanes, the signal from *miR-8* microRNA in the total carcass RNA is demonstrated at lower signal intensity.

The accumulation of fragments in Zuc endonuclease mutants suggests that the formation of the fragments is unlikely to be connected with promiscuity of endonucleases integral to the primary piRNA processing mechanism of piRNA biogenesis. This statement is supported by the fact that, while the piRNA pathway functions in gonads, when compared with the equivalent amount of total RNA from heterozygous ovaries, the rRNA fragments are usually more readily discernible in somatic tissues (flies without ovaries called carcasses) (Figure 4), appearing to contain a smaller set of fragments, which appears as mostly a subset of those in mutant ovaries and has the potential to accumulate in comparable quantities (Figure 4). It should be taken into account that carcass RNA contains RNA from a multitude of somatic tissues, which makes it difficult to quantitatively compare with ovarian RNA. However, this result shows that at least some of the proteins that provide the activity for the accumulation of rRNA fragments appear to be ubiquitous, rather than gonad-specific. Thus, Piwi or the primary piRNA pathway could be a part of a system protecting the especially vulnerable ovarian cells from accumulation of RNA fragments.

It should be noted that mutations of all the analyzed genes (*piwi^2/Nt^*, *armi*, *zuc*, *spn-E*, *aub*), as well as their germline knockdowns, are known to cause sterility at the stage of embryonic death, which is typical of piRNA pathway defects [46,87,90]. To check that fragment accumulation is not a result of embryonic death, the onset of which could be thus manifested in the ovaries, the embryos, which die before the beginning of first instar larval stage (~22 h [93,94]) due to a failure in piRNA silencing, were divided into young and old groups (Supplementary Figure S2). According to preliminary data, in the young group 0–2.5 h old, rRNA fragments are similarly observed in control embryos of the *nos-GAL4* driver line and in embryos laid by *nos-GAL4*-driven germline-specific *piwi* knockdown (*piwiGLKD*) [95] mothers. In the old group 2–24 h old, the death of the knockdown embryos primarily manifests itself in the absence of synthesis of the pre-rRNA, as well as in the smearing of the fragments (Supplementary Figure S2). Probably in the embryos from mothers with piRNA pathway disruption, zygotic rRNA transcription is never turned on. Thus, fragment accumulation is not exacerbated by embryonic death. Moreover, this is disproved by the difference with respect to rRNA fragment accumulation between the mutations in primary and ping-pong components of piRNA silencing (Figure 3).

### 2.3. The Piwi Mutation Induces a Processing Defect of Drosophila 2S rRNA, Orthologous to the 3′-End of Eukaryotic 5.8S rRNA

**Figure 5:**
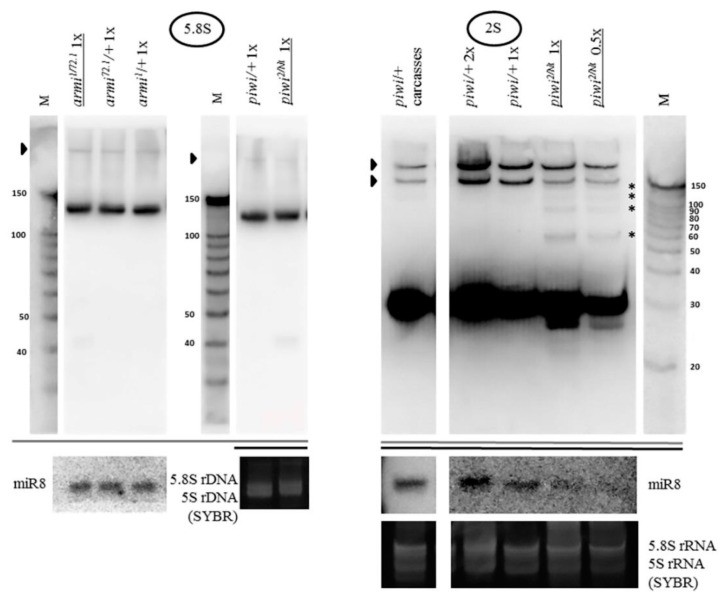
The *piwi* mutation induces a processing defect of *Drosophila* 2S rRNA orthologous to the 3′-end of eukaryotic 5.8S rRNA: In *piwi* mutants, precursors of 2S rRNA accumulate with approximate sizes of 60, 90, 120, and 150 *n*. (on the right, marked by asterisks), detected with a probe to 2S rRNA 30 *n*. long. Precursor sizes of 5.8S rRNA appear to be unaffected (on the left, which are several lanes from the blots shown in Figure 3A, demonstrated here at lower signal intensity). Wild-type rRNA precursors are marked with black arrowheads.

The rRNA fragments are usually apparently shorter than mature rRNA molecules (Figure 1B). rRNA precursor signals in the *piwi* mutant ovaries generally appear to be similar to controls in their sizes and their ratio to mature species (Figure 1B for 18S rRNA and 28S rRNA, Figure 5 for 5.8S rRNA, Figure 2 for 5S rRNA). However, an obvious change in precursor sizes is observed for 2S rRNA, a separate fifth rRNA found in *Diptera* [96], with a probe to the entire 2S rRNA (Figure 1A, Figure 5). Importantly, this effect is not apparent in carcasses (Figure 5) and ping-pong component *spn-E* mutants (data not shown). While in other organisms 5.8S rRNA and 2S rRNA are represented by a single molecule [20], in *Drosophila* 2S rRNA is split from the 3′-terminus of 5.8S rRNA by discarding a 28 *n*. spacer from a reported precursor of 181 *n*. [97]. In the *Drosophila* cytoplasm, 5.8S rRNA and 2S rRNA are already detected as separate mature rRNAs of 123 *n*. and 30 *n*. in length, respectively [98]. In *piwi* mutants, a probe to 2S rRNA reveals the accumulation of several precursors of 2S rRNA with sizes of approximately 60, 90, 120 and 150 *n*. (Figure 5).

Since the processing alterations of 5.8S rRNA still combined with 2S rRNA into a transcript of 181 *n*. or longer do not seem to be apparent (Figure 5), and the observed sizes of the 2S precursors, even assuming the 28 *n*. spacer retained, are shorter than 181 *n*., it is possible to assign the processing defect to the 3’ end of 2S rRNA, already split from 5.8S rRNA. Religation of 5.8S and 2S after excision of the 28 *n*. spacer (as a result of which the size of the transcript would be 153 *n*.) was not shown to exist [20], therefore, the accumulated fragments cannot be explained by a defect in the maturation of the ends of this putative fused precursor in the ITS1 or ITS2 regions, in which cases the precursors would be longer than the ones observed (see Figure 1A). If this defect were related to the maturation of the 5’ end of 2S rRNA, namely, the excision of the 28 *n*. spacer between 5.8S rRNA and 2S rRNA, then it would lead to the accumulation of a precursor 58 *n*. long, with a band approximately this size appearing to be observed; but it does not explain the accumulation of precursors of longer sizes. Thus, in *piwi* mutants the maturation of the 2S rRNA 3’ end in the ITS2 region, located between 2S rRNA and 28S rRNA (see Figure 1A), appears to be impaired resulting in extensions of variable length. This defect resembles the one that lead to the discovery of the exosome as the complex performing the maturation of the 3’ end of 5.8S rRNA in yeast [99]. It is worth noting that the maturation defects of *Drosophila* 2S rRNA have been reported in exosome subunit knockdowns in embryonic S2 tissue culture cells [97], while in germline tissues, to the knowledge of the author, they have not been previously demonstrated.

The excision of ITS2 and maturation of 5.8S rRNA are some of the most complicated pre-rRNA processing events in eukaryotes involving a combination of exo- and endonucleolytic activities, to which the ubiquitous nuclear exosome makes an important contribution [100], including its 3′-5′-exo-/endonuclease Rrp44/Dis3 and 3′-5′-exonuclease Rrp6 subunits, functioning after the initial Las1 endonucleolytic event splitting the pre-rRNA in ITS2 [101]. When the synthesis of ribosomal RNA proceeds normally, the activity of the exosome is terminated close to the mature 3′-end of rRNA. In the absence of the exosome components, some other activity is able to partially digest the 5.8S rRNA precursor, producing a ladder of intermediates [102]. ITS2 processing in *Drosophila* is poorly studied. In *Drosophila* S2 cells, similarly to the well-studied yeast [103] and mammalian [100] systems, Rrp6, and especially Dis3, causes accumulation of fragments longer than 2S rRNA [97]. The fact that the processing defect in the present work is detected only for 2S rRNA, but not for 5.8S rRNA (Figure 5), may mean that *Drosophila* 2S rRNA may be able to split (but not necessarily always, since the mature joint 181 *n*. precursor is detectable [97]) from a common precursor with 5.8S before the completion of ITS2 processing. That means that (at least) the beginning of the processing in the 28 *n*. spacer can be hypothesized to sometimes precede ITS*2/2*S rRNA 3′-end processing and 2S rRNA maturation. Although alternative pathways of rRNA maturation are widely known [104,105], this statement requires further studies.

Due to its classic distinctiveness, the effect of the accumulation of 2S rRNA precursors with 3’ extensions in *piwi* mutants could be hypothesized to be attributed to a defect in protein function most likely acting in the nucleus, responsible for 5.8S rRNA maturation in other organisms studied, including the exosome or exosome-associated/exosome-targeting proteins, participating in RNA surveillance, described in detail in Section 3. It is worth noting that RNA processing and RNA surveillance are frequently performed by the same nucleases. Probing of pre-rRNA maturation intermediates by exonucleases has been proposed to serve the dual function of producing mature rRNAs and suppressing suboptimal processing pathways during ribosome biogenesis by converting the processing function to the turnover function [106,107].

### 2.4. The Accumulation of rRNA Fragments in Piwi Mutants Is Accompanied by the Accumulation of Antisense RNA, Shown for 5S rRNA

**Figure 6:**
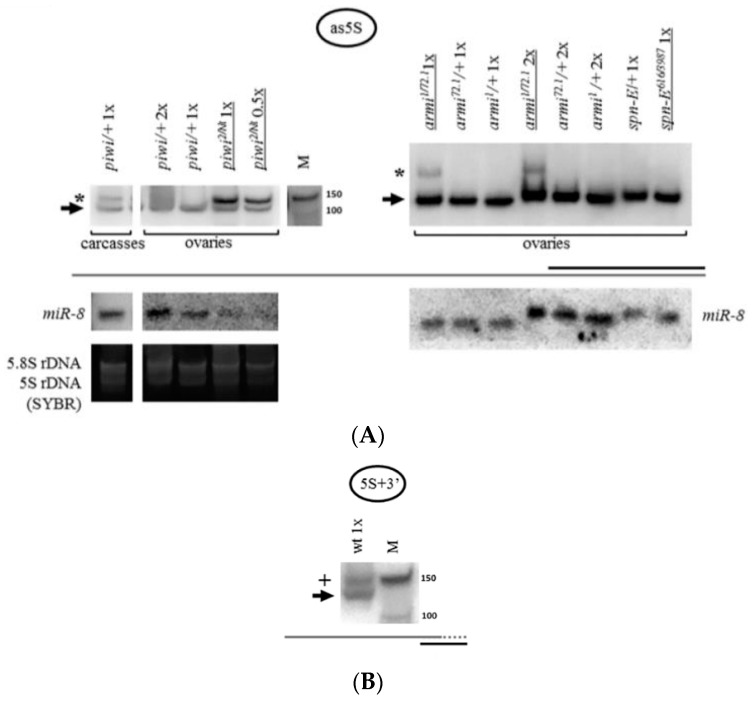
The accumulation of 5S rRNA fragments is accompanied by the accumulation of antisense 5S rRNA: (**A**) In *piwi* and *armi* mutant ovaries and in control carcasses the accumulation of an antisense 5S rRNA transcript is observed. A sense probe homologous to the 3′-end of mature 5S rRNA was used. The antisense transcript is marked with an asterisk. The black arrow marks an artifactual signal from the mature 5S rRNA molecule which is observed due to the potential for duplex formation of the probe with parts of the sense 5S rRNA molecule and the high quantity of the latter in the specimen; (**B**) The antisense 5S rRNA transcript resembles in apparent size the complete immature pre-5S rRNA transcript. An antisense probe homologous to the 3′-end of pre-5S rRNA was used. This transcript (marked with a plus sign) is characterized by a 14 *n*. 3′-extension (depicted by the addition of a dotted line to the grey coarse schematic representation) and is normally observed in wild-type ovaries. The wt genotype did not contain a mutation in *piwi* (*nos-GAL4* driver line). The signal from the mature 5S rRNA molecule (black arrow) is also observed, detected due to complementarity with approximately half of the probe.

The endosiRNA-sized fragments of 5S rRNA present in *piwi* mutants (Figure 2) are difficult to explain based on the secondary structure of the molecule [108], because 5S rRNA itself does not form a perfect duplex of ~21 *n*. at the 5′-terminus that could be processed by Dicer2. Hence, the existence of double-stranded RNA may be hypothesized, formed by base-pairing of the 5S rRNA molecule with an antisense transcript. Indeed, such antisense RNA was found (Figure 6A), detected with a probe to the region of the 3′-end of the mature rRNA. The antisense transcript accompanies 5S rRNA fragment accumulation observed in *piwi* and *armi* mutant ovaries and in wild-type carcasses of *Drosophila*, where a subset of rRNA fragments is also observed, and is not detected in the ping-pong component *spn-E* mutant organs (Figure 6A). In the present work antisense rRNA has been checked for just one rRNA type, 5S rRNA, and whether other rRNAs produce antisense transcripts in this system remains to be determined. Although non-templated polyadenylation cannot be ruled out, it is interesting that the antisense transcript resembles in size the complete 5S rRNA precursor transcript produced by RNA polymerase III (135 *n*.) before its maturation to 120 *n*. [109] (Figure 6B). The report of the possibility of RNA polymerase II enrichment at the complete rRNA repeat [29] might allow to predict this transcript to be produced by RNA polymerase II and encompass the complete transcription unit. Thus, normally Piwi is required for antisense accumulation prevention at the 5S rRNA genes.

## 3. Discussion

### 3.1. Overview and Significance of the Discovered Effects of Piwi Dysfunction on rRNA Metabolism

In this work it was shown that mutations in the gene coding for the predominantly nuclear Argonaute protein Piwi and in the genes of other primary piRNA processing components of the piRNA pathway preventing its entrance into the nucleus, but not ping-pong components, cause a significant accumulation of fragments of all five rRNAs in *Drosophila* ovaries. Thus, the relatively evolutionarily recently acquired animal piRNA pathway [110] can affect the ancient ribosome machinery. Being mostly shorter than mature rRNAs, the rRNA fragments appear to represent dysfunctional transcripts the accumulation of which constitutes a significant problem for cells [15]. Maintaining their timely elimination could be particularly important in the cells responsible for embryo development and supply during oogenesis. Additionally, in these cells the piRNA pathway operates, which is supposedly able to process aberrant transcripts as precursors of piRNAs and, thus, lead to undesirable suppression of homologous genes. Although effects of piRNA silencing defects on other RNAs require further studies, at this point it is possible to assume that there could be a protective gonad-specific system in *Drosophila melanogaster* for preventing the accumulation of rRNA fragments.

Furthermore, the *piwi* mutation appears to interfere with the processing of the ortholog of the 3′-end of eukaryotic 5.8S rRNA, represented in *Drosophila* by 2S rRNA. The processing defect has not been previously demonstrated in *Drosophila* germline tissues and allows to suspect the existence of an alternative rRNA processing pathway, in which dipteran 5.8S rRNA could be split from 2S rRNA before 2S rRNA 3′-end maturation completion.

Moreover, the 2S rRNA processing defect and rRNA fragment accumulation in *piwi* mutants is accompanied by accumulation of antisense rRNA shown for the 5S rRNA transcript. In the literature, an antisense lncRNA homologous to 5S rRNA and transcribed by RNA polymerase II, termed 5S-OT, has been described in mammals, including germline tissues [111]. 5S-OT with a size of over 200 *n*. has been shown to be formed in the sense direction to 5S rRNA in the *Drosophila* soma [111]. Thus, the accumulation of the antisense RNA observed in this work cannot be considered merely as an activation of lncRNA described earlier. To the knowledge of the author, the present work is the first report demonstrating the existence of an antisense 5S rRNA in *Drosophila*.

It is likely that 5S rRNA and the other rRNAs encoded by the 18S/28S rDNA locus undergo similar changes in *piwi* mutants, because 5S rRNA is connected with other ribosomal rRNAs, at least via the cross-talk of RNA polymerases, jointly producing ribosomal components [112]. 5S rRNA can even have a leading or regulatory role among rRNAs, in accordance with its function in the 5S rRNA–Rpl5–Rpl11 complex activating p53 for apoptosis [113,114]. Part of the importance of 5S rRNA, capable of being redirected as part of this complex from assembly into nascent ribosomes to p53 regulation as a consequence of impaired ribosome biogenesis, lies in serving as a guarantee that all the processes of ribosome biogenesis are correct [114]. Any disruption in ribosome biogenesis is nucleolar stress (also referred to as ribosomal stress) [115,116]. Perturbations in ribosome biogenesis can cause disruptions in nucleolar integrity. From this point of view, the nucleolar disruption found in *Drosophila dicer2* and *piwi* mutants in the soma can reflect nucleolar stress, demonstrated before the coining of the term [73]. The results of the presented work on 2S rRNA maturation can lead to the conclusion that the *piwi* mutation induces nucleolar stress in the gonads.

### 3.2. Hypotheses of Piwi Functioning in rRNA Metabolism

#### 3.2.1. Possible Direct Roles of Piwi

Several hypotheses to explain the observed effects on rRNA metabolism can be envisioned. Among the direct ways how Piwi dysfunction can cause the observed effects, could be ectopic dual-strand piRNA cluster formation at repetitive rDNA. A recent study found that depletion of Piwi from developing germ cells during a window in embryogenesis leads to deterioration of the chromatin state at proper piRNA clusters in adult ovaries [117], opening a possibility for ectopic cluster induction. This hypothesis is consistent with the observation of antisense RNA, an outstanding feature of sequences going into the piRNA pool [48], accumulated on 5S rRNA and modified rRNA processing, similarly to the rewired termination and splicing effects reported at dual-strand piRNA clusters [53]. Another direct way how Piwi can normally prevent the observed effects is by being a player in nuclear RNA surveillance in *Drosophila* ovaries involved in proper rRNA functioning. In line with this, there are reports of similar direct roles of small RNA pathway components: Dicer directing antisense rRNA transcription termination in yeast [29], Argonaute proteins regulating lncRNA abundance in mice [118] or acting as an RNA silencing suppressor in *C. elegans* [119].

The three main phenomena of the Piwi dysfunction phenotype observed in this work have been described in the literature in different organisms in cases that are not connected with piRNA silencing. The intersection of their causes can help to deduce the more likely indirect ways by which Piwi dysfunction can lead to the observed effects.

#### 3.2.2. rRNA Fragments

In the literature, long rRNA fragments in eukaryotes were reported to be generated in response to viral infection by the mammalian RNAse L system [120] absent in *Drosophila*, to be present under oxidative stress and apoptotic conditions [121,122], as well as in the case of a number of mutations or depletions of ribosome biogenesis factors [123], of exosome and exosome-targeting components alone [97,124] and combined with stress/environmental insults [97,125,126]. Moreover, a change in environmental conditions can be expected to cause rRNA fragment accumulation, since recently it has been shown to lead to surveillance pathway downregulation [127,128]. One could envision two principal modes of fragment accumulation—via RNA initiation or RNA stability. Being smaller in size than the mature transcripts, the rRNA fragments seem to derive from mature rRNAs present in the cytoplasm, which could be driven, among others, by the ping-pong Argonautes or the endosiRNA pathway, falling into the category of RNA stability. However, the limitation of the analysis presented in this work is that probes to mature rRNAs, the most abundant RNA molecules in cells, can sometimes prevent the detection of subtle effects on precursor rRNAs present in the nucleus. In the literature, rRNA fragments of small RNA sizes, that were shown to exist in normal tissues in a number of organisms studied, are usually considered to originate from mature rRNA transcripts or pre-rRNA [81]. The potential mechanisms of fragment accumulation via RNA stability can be divided into the degradation into fragments not usually formed and the stabilization of fragments usually eliminated, a distinction which requires further study.

#### 3.2.3. Antisense rRNA

Antisense RNA accumulation, studied in detail for fission yeast noncoding RNAs, has been shown to be controlled by three RNA processing pathways [18,129] – nuclear exosome, nuclear Dicer, and cytoplasmic Xrn1. Antisense RNA formation has also been shown to be characteristic of piRNA cluster formation [36,48,130]. Speaking of cases where rRNA is affected, antisense transcripts can be present in the form of lncRNAs [111,131,132], resulting from defects in transcription termination at sites of replication stress [29], in cases of RNA surveillance [24,26], RNAi and chromatin impairment [24,79,133], and rRNA processing and modification defects [77,134], and can be a result of viral infection [135], physiological conditions and environmental stimuli [134], as well as double-stranded DNA breaks [136].

#### 3.2.4. 5.8S rRNA Maturation

Taking the most specific defect, the impairment of 2S rRNA maturation, which in most other organisms is 5.8S rRNA maturation, could be attributed to a defect in the exosome [97,99,102,137,138] and serum starvation stress effect on the exosome [128], in exosome-associated/exosome-targeting components Mtr4 [100], Nop53 [139], Mpp6 [140,141], Rrp47 [138,142], or proteins coordinating 5′- and 3′-ends of 5.8S rRNA maturation Las1 and even the 5′–3′ exonuclease Xrn2 [143] also reported to be involved in RNA surveillance, as well as human microRNA/RNAi components Dicer and Ago2 and microRNA machinery component Drosha [28,144].

Speaking of an indirect role of Piwi in the observed phenomena, the intersection of the causes for the discovered effects can allow the hypothesis that *piwi* dysfunction can lead to defects in the RNA surveillance machinery or RNAi, as the most likely candidates. Why fragment accumulation and extended 2S rRNA accumulation concern a small fraction of rRNA remains unknown, however, this resembles both the exosome mutants [145] and microRNA/RNAi depletion [28], and could be relevant for a subset of rDNA repeats (for example, active or inactive units, units carrying or not carrying R1 and R2 transposable element insertions in 28S rRNA [70]).

#### 3.2.5. Defects in RNAi as a Candidate for the Cause of the Piwi Dysfunction Phenotype

RNAi seems less likely as a potential cause of defects observed in *piwi* mutants, because RNAi, unlike *piwi* dysfunction, additionally causes 5.8S rRNA 5′-extensions [28] and has not been linked with 5.8S rRNA processing in organisms other than human somatic HeLa cells where RNAi has evolutionarily been merged with the microRNA machinery to regulate endogenous gene expression [27,47,146].

The connection of RNAi component Dicer with antisense rRNA accumulation has been studied in detail in fission yeast [29]. Dicer protein participates in the termination of antisense RNA polymerase II transcription on rDNA accompanied by the formation of short RNAs of both polarities. A violation of transcription termination is known to result in the absence of a change or a slight decrease in the level of transcripts, as shown for protein-coding genes [29]. At the DNA level, failure in termination leads to a decrease in the number of rDNA repeats, reflecting the destabilization of the genome [29]. This is interpreted as a consequence of replication stress due to a conflict of transcription and replication, when the paused replication forks generated by a collision with RNA polymerase II can only be resolved by homologous recombination. Although this was found to be an Ago-independent process [29], in human cells double-stranded RNA formation prevention at rDNA is thought to involve Ago1 [147]. It is possible to hypothesize a role for Piwi in releasing RNA polymerase II, maybe via aiding Dicer, at transcription-replication collisions.

To the knowledge of the author of this work, RNAi has not been previously linked to the accumulation of rRNA fragments. For this purpose, genetic crosses were carried out to produce double mutant flies in *piwi* and *ago2*. According to the preliminary data, accumulation of the fragments was observed both in ovaries of *piwi^2/Nt^* mutants (heterozygous for *ago2*^414^) and to a lesser extent in ovaries of *ago2^414/414^* mutants [45] (heterozygous for a mixture of *piwi^2^* and *piwi^Nt^* mutations) (Supplementary Figure S4). The progeny of the cross, combining *piwi* and *ago2* mutations, had ovaries with disturbed morphology, up to complete absence in the double mutants. However, mutants of the *ago2* line itself showed no accumulation of rRNA fragments (data not shown). This preliminary result speaks in favor of a genetic interaction between the two genes. It was suggested that in *Drosophila* the specialization of Argonaute proteins in the endosi- or piRNA-silencing pathways can be relative [148]. Additionally, it is known that RNAi in *Drosophila* ovaries does not function very effectively, judging from data on transgenic constructs [149]. Thus, it is possible that in *Drosophila* ovaries, possessing both pathways, piRNA silencing may play a leading role in this phenomenon, which requires further studies.

#### 3.2.6. Defects in RNA Surveillance as a Candidate for the Cause of the Piwi Dysfunction Phenotype 

A more likely assumption is that the *piwi* mutation could be accompanied by a defect in RNA surveillance carried out by machinery that overlaps with that of rRNA processing and capable of causing all of the observed effects. There are reports of surveillance knockdowns and mutations causing piRNA like effects, shown for exosome and exosome-interacting complexes [95,150,151,152,153,154,155]. Moreover, RNA surveillance has been very recently found in *Drosophila* to be required for clearing RNA from the chromatin of transposable elements in order to maintain effective piRNA silencing [154], as proposed earlier based on yeast studies [156,157]. In this work it was shown that the exosome-interacting surveillance CCR4-Not complex localization is Piwi-dependent and CCR4 interaction with Piwi was detected, however, small RNA levels are not changed upon CCR4 depletion. It is tempting to speculate that the present study supports a model in which Piwi could control surveillance on the tandem rRNA repeats by mediating the recruitment of the exosome or exosome-interacting proteins to rRNA.

In yeast, the absence of surveillance (recently found to be directed at Argonaute-bound small RNAs [80]) leads to spurious siRNA production from endogenous genes, including rRNA, possibly threatening ribosome production, because surveillance and silencing have been reported to compete for substrates [26], which is also shown in plants [158]. Thus, dysfunctional RNA surveillance can cause alterations in small RNA profiles. In addition to surveillance components of the exosome-targeting cofactor TRAMP in yeast [26], cases of small RNA profile alteration reported in the literature include defects in yeast exosome Rrp6 subunit [34,159], in RNA-binding protein La functioning as a gatekeeper ensuring correct tRNA maturation and protecting the microRNA pathway from potentially functional tRNA fragments in humans [160], RNAi mutants in *C. elegans* [161,162] and in *Drosophila* when small RNAs antisense to rRNA and ITS1 accumulate in the total RNA and microRNA pathway component Ago1 precipitates, a system described by the authors to reflect the endosiRNA pool [79], as well as the piRNA component Tdrd1 defect in mice [67]. In the latter case, due to this defect, causing the disruption of nuclear import of Argonaute Miwi2, the remaining cytoplasmic Argonaute protein Mili accumulates short RNAs originating from 5S rRNA [67], similarly to the piRNA-like sized 5S fragments observed in the present work. The ping-pong system remains functional, which is also observed when Miwi2 is knocked out [163]. This was interpreted as the role of Tdrd1 in limiting the range of piRNA silencing targets [67]. However, another interpretation could be that the correctly located mainly nuclear Argonaute itself could be important in safeguarding the short RNA profile, whereas spurious piRNA generation could be accomplished by the ping-pong machinery.

Thus, dysfunctional RNA surveillance can cause alterations in small RNA profiles and dysfunctional nuclear Argonautes can be hypothesized to cause alterations in small RNA profiles. Then, dysfunction of nuclear piRNA Argonaute Piwi absence could be connected with a defect in surveillance. It could be that in monopartite small RNA silencing systems, like that of yeast, where surveillance and RNAi compete, in surveillance mutants RNAi wins the competition. In bipartite systems, like that consisting of primary processing and ping-pong in *Drosophila*, primary processing and ping-pong are in competition that ping-pong wins [61] in case of mutations in nuclear Argonautes. Ping-pong in *piwi* mutants is functional [88,89]. In this case, perhaps primary processing can be hypothesized to be teamed with surveillance and possibly counteract cytoplasmic pathways including the remaining ping-pong Argonautes. Since spurious piRNA generation involves rRNA, this might highlight the importance of functional nuclear Argonautes in guarding tandem repeats.

Surveillance defects, in turn, can cause stress, like nucleolar stress described above. At the same time, surveillance defects are themselves also exacerbated upon stress. The *piwi* mutation can present a source of stress, because transposable element mobilization is known to cause DNA damage signaling activation [46]. As for stress concerning rDNA, but not directly connected with transposable elements, rDNA is considered the most unstable region in the genome due to repetitive nature and high transcriptional activity [164] and could represent an even more fragile region in polytene cells, including nurse and follicle cells of *Drosophila* ovaries. Transcription of rDNA takes place in the S phase accompanying replication [165]. Failure of replication forks and repair are observed in polytene cells quite often [166] and the cells experience constant genotoxic stress associated with underreplication of heterochromatic DNA [167]. However, normally genotoxic stress response in polyploid cells is suppressed [168].

#### 3.2.7. Possible Downstream Events of the Piwi Dysfunction Phenotype

The following downstream events can be envisioned as a result of antisense rRNA accumulation. It can cause replication stress from transcription-replication conflicts, in turn causing replication fork collapse as described above [29], or from the accumulation of R-loops [169]. This will eventually lead to DNA damage, including DNA double-stranded breaks, in tandem rDNA repeats [170]. DNA double-stranded breaks activate DNA damage signaling. The signaling can end up in repair, cell cycle arrest or apoptosis [171]. Since in polytene cells there is normally no apoptosis or cell cycle arrest [168], the signaling is likely to promote repair, which can be carried out by homologous recombination or by nonhomologous end-joining. In rDNA it is mostly accomplished by homologous recombination [172].

Recombination-mediated repair of DNA double-stranded breaks in the rDNA can result in a loss of repeat integrity [173,174], in accordance with which most of the causes of antisense RNA accumulation are accompanied by genome instability in rDNA tandem repeats in different organisms [29,73,156,175,176,177,178,179,180,181]. Indeed, there is indirect evidence that *piwi* mutants can potentially manifest the same rDNA instability phenomenon (formation of eccDNA) in somatic cells [73], where the piRNA pathway is usually inactive (see Introduction). This allows the author of this work to assume that the *piwi* mutation could accumulate eccDNAs containing tandem repeats in the ovaries where Piwi can function as part of the complete piRNA pathway. According to earlier studies, tandem repeats, including rDNA, are prone to formation of eccDNA, whereas dispersed repeats form such circles to a much lesser extent [182,183]. Thus, the phenomena described in the present work might point to a special piRNA pathway function in antisense RNA prevention and stability of tandem repeats in the gonads, as opposed to its much more well-studied function in dispersed transposable element repeat silencing.

Since a subset of the rRNA fragments, antisense rRNA (this work), and rDNA eccDNA [182] normally exist in the soma, polyploidy could be the phenomenon that could make the response different in ovaries and carcasses containing just some polyploid tissues. The *piwi* effect on nucleolus disruption in *Drosophila* soma appeared more prominent in polytene salivary gland cells, in comparison with diploid somatic cells [76]. Thus, it is possible that Piwi and the whole primary piRNA pathway may contribute to the stability of the genome in polyploid cells of the gonads, characterized by complexities in the separation of replication and transcription.

### 3.3. Summary

In conclusion, the nuclear piRNA pathway component Piwi regulates rRNA fragment accumulation, 2S rRNA processing, and 5S rRNA antisense transcript accumulation in the ovaries, possibly directly or by functioning together with RNAi components or, more likely, the RNA surveillance machinery. Piwi dysfunction can be hypothesized to be accompanied by spurious piRNA generation, replication stress, and genome instability in the rDNA repeats in gonad polytene cells. It is essential to prevent these effects in gonad cells which are responsible for embryo development, where it is critical that ribosome production should not be threatened. Of note, the piRNA pathway is clinically important, since its disruption causes sterility in *Drosophila* and mammals, and ectopic expression of its proteins has been shown to correlate with cancer [75].

This work continues to uncover new rRNA-related functions of the Piwi protein and piRNA pathway, as well as assumes their possible role in the regulation of tandem repeats that can be different from their well-known function in dispersed transposon repeat suppression. The continuation of this work will strive to fill the void in the knowledge of the interaction of the piRNA pathway and rRNA metabolism in *Drosophila*, which will allow the implementation of the power of *Drosophila* genetics in future studies of this exciting topic. 

## 4. Materials and Methods

### 4.1. Strains of Drosophila melanogaster

Flies were maintained at 25 °C on standard medium. For embryo collection, flies were maintained at room temperature. In order to analyze the *piwi* phenotype, transheterozygous *piwi^2/Nt^* females and their female siblings (a mixture of *piwi^Nt^*/+ and *piwi^2^*/+ flies) from the progeny of the same cross were compared. The following mutant flies were used: *piwi^Nt^*, *piwi^2^*, and *piwi^3^* [51]; *armi^1/72.1^* [86]; *zuc^HM27^/Df(2L)Prl* [87]; *spn-E^616/3987^* [90,184]; *aub^QC42/HN2^* [91]; *ago2^414/414^* [45]. The respective heterozygous flies were used as controls. To generate flies with *piwi* knockdown in the germline (*piwiGLKD*), *piwi* RNAi flies VDRC #v101658 (*w[1118]*; *P{KK105350}VIE-260B*) were mated with the *nos-GAL4* driver line Bloomington #25751 (*P{w[+mC]=UAS-Dcr-2.D}1*, *w[1118];* *P{w[+mC]=GAL4-nos.NGT}40*) [95].

### 4.2. RNA Isolation

Total RNA was extracted from 30–50 dissected ovaries of 0–6 day-old flies using Trizol reagent (Invitrogen, Carlsbad, CA, USA) according to the manufacturer’s instructions. Embryos were collected and dechorionated in 50% bleach prior to RNA extraction. RNA quantity was measured using NanoDrop 1000 spectrophotometer (Thermo Fischer Scientific, Wilmington, DE, USA).

### 4.3. Northern Blot Hybridization

Various quantities of total RNA in equal amounts for mutant and control ovaries were separated by electrophoresis. For long RNAs, electrophoresis of 1, 2, and 4 mkg total RNA was carried out in a denaturing formaldehyde 1% agarose gel in MOPS buffer in accordance with the guidelines [185]. Blotting was performed with upward capillary transfer in alkaline conditions [185] or downward capillary transfer under neutral conditions [186] to the Hybond-N+ membrane (Amersham, Amersham, UK) with subsequent fixation of RNA under UV. For small RNAs, electrophoresis of 10 and 20 mkg total RNA was done in denaturing 10 or 20% polyacrylamide gel with 7 M urea in 1× TBE buffer and electroblotting was performed in an XCell II blot module (Invitrogen, Carlsbad, CA, USA) with subsequent fixation of RNA under UV. The following size markers were used: Ambion Decade marker (10, 20, 30, 40, 50, 60, 70, 80, 90, 100, 150 *n*.) and Ambion Millenium marker (500, 1000, 1500, 2000, 2500, 3000, 4000, 5000, 6000, 9000 *n*.) (Invitrogen, Carlsbad, CA, USA). Hybridization was performed at 37 °C in Church buffer with complementary DNA oligonucleotides labeled with [gamma-32P]UTP by polynucleotide kinase in Buffer B according to the manufacturer’s instructions (Thermo Fischer Scientific, Wilmington, DE, USA) with subsequent washing by 2xSSC/0.1% SDS twice and 1XSSC/0.1% SDS once. Visualization was performed using a Typhoon FLA9500 (GE Healthcare, Chicago, IL, USA) phosphoimager. Usually the same filter was hybridized consecutively with different probes and stripped by boiling 0.1% SDS. Before transfer, RNA in the gels was visualized by SYBR Green II (Invitrogen, Carlsbad, CA, USA) on Bio-Rad Gel Doc gel documentation system (Bio-Rad, Hercules, CA, USA). In addition to equal amounts of RNA analyzed, *miR-8* microRNA and ribosomal protein *rp49* transcripts were used as loading controls. Northern blot for each genotype was performed at least twice with independently isolated RNAs, except for supplementary material experiments performed once. To design the probes, GenBank accession numbers of the following DNA sequences were used: rRNA–M20107.1, 5S rRNA–J01122.1. The following probes were used:

5S end AGGCCAACAACACGCGGTGTTCCCAAG (27 *n*.)

18S end TAATGATCCTTCCGCAGGTTCACCTAC (27 *n*.)

5.8S end CAGCATGGACTGCGATATGCGTTCAAA (27 *n*.)

2S TACAACCCTCAACCATATGTAGTCCAAGCA (30 *n*.)

28S end TCGAATCATCAAGCAAAGGATAAGCTT (27 *n*.)

5S start TGTATTCAGC3GTGGTATGGTCGTTGGC (27 *n*.)

5S mid CGACGCTGCTTAATTTCGGTGATCGGACGAGA (32 *n*.)

5S end AGGCCAACAACACGCGGTGTTCCCAAG (27 *n*.)

5S end sense CTTGGGAACACCGCGTGTTGTTGGCCT (27 *n*.)

5S 3′ AAAAAGTTGTGGACGAGGC (20 *n*.)

5.8S mid GTCGATGTTCATGTGTCCTGCAGTTCACACGATGACGCACAG (42 *n*.) [105]

*miR-8* GACATCTTTACCTGACAGTATTA (23 *n*.)

### 4.4. Real-Time RT-PCR

After RNA isolation with Trizol reagent, RNA was reprecipitated in 3.3 M LiCl, centrifuged, and dissolved in water, DNAse-treated (DNAse I, Thermo Fischer Scientific, Wilmington, DE, USA). 1 mkg of total RNA was used to synthesize cDNA in RT+ and RT– reactions for each genotype using random hexamers with Mint reverse transcriptase (Evrogen, Moscow, Russia). Real-time PCR was performed with SYTO13 intercalating agent and HS Taq DNA polymerase (Evrogen, Moscow, Russia). For calibration the *piwi^2/Nt^* genotype was used. Thermal cycling consisted of 5 min at 95 °C, followed by 45 cycles of denaturation (94 °C, 20 sec), annealing (64 °C, 20 sec), extension (72 °C, 20 sec), and a final extension of 5 min at 72 °C. RT– reactions did not show significant PCR signals. One biological replica per genotype was analyzed, in three technical replicates of real-time PCR. *Adh* transcript was used as a loading control.

The following primers were used: for *HeT-A* retrotransposon Het-s2 (CGCAAAGACATCTGGAGGACTACC) and Het-as2 (TGCCGACCTGCTTGGTATTG) [51], for *Adh* AdhRI_s (GCCTGCGTACATAGCCGAGAT) and AdhRI_as (GCTCCGTTAGTTGTTGGTTTCC). 

## Supplementary Materials

Supplementary materials can be found at https://www.mdpi.com/1422-0067/21/3/1119/s1.

## Abbreviations

eccDNA	Extrachromosomal circular DNA
endosiRNAs	Endogenous small interfering RNAs
ETS	External transcribed spacer
ITS2	Internal transcribed spacer 2
lncRNA	Long non-coding RNA
NTS	Nontranscribed intergenic spacer
piRNAs	Piwi-interacting RNAs
rDNA	Ribosomal DNA
RNAi	RNA interference
rRNA	Ribosomal RNA
siRNA	Small interfering RNAs
