# Supplementary material for: Nuclear Argonaute Piwi Gene Mutation Affects rRNA by Inducing rRNA Fragment Accumulation, Antisense Expression, and Defective Processing in Drosophila Ovaries
Source: Int J Mol Sci. 2020 Feb 7;21(3):1119. doi: 10.3390/ijms21031119 (PMC7037970; doi:10.3390/ijms21031119)
Supplement: Supplementary file 1 [file ijms-21-01119-s001.pdf]

Supplementary Material

to article:

Nuclear Argonaute Piwi Gene Mutation Affects rRNA by Inducing rRNA Fragment Accumulation, Antisense Expression and Defective Processing in *Drosophila* Ovaries.

Anastasia D. Stolyarenko

Institute of Molecular Genetics, Russian Academy of Sciences, 2 Kurchatov Sq., Moscow 123182, Russia

For correspondence: stol@img.ras.ru

Supplementary Fig. 1

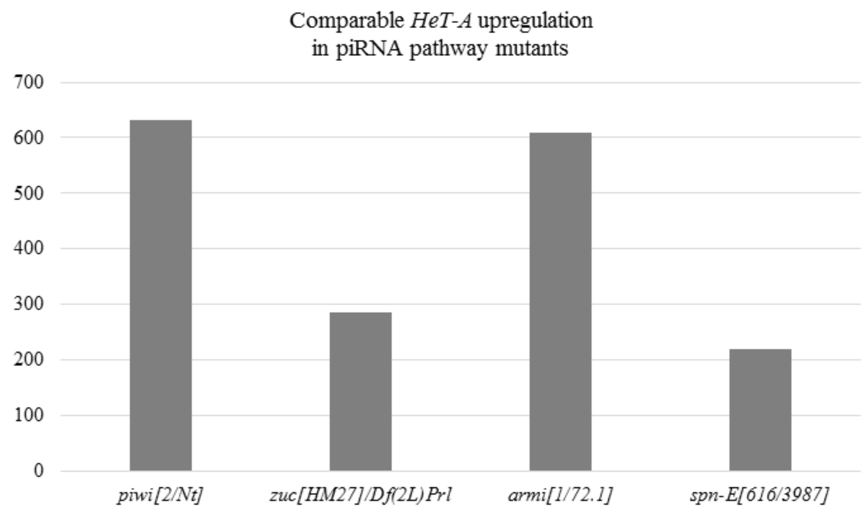

Supplementary Figure S1. Checking *piwi* and other mutations used in this work for transposable element upregulation in *Drosophila melanogaster* ovaries.
Fold-change of transcript abundance for the model transposable element *HeT-A* above that of control heterozygotes is shown for each mutant. A comparable effect of upregulation is observed in the mutant ovaries analyzed. The experiment was performed once with three technical replicates. The alcoholdehydrogenase *adh* gene, a constitutively transcribed gene normally not associated with the piRNA pathway, was used as a loading control.

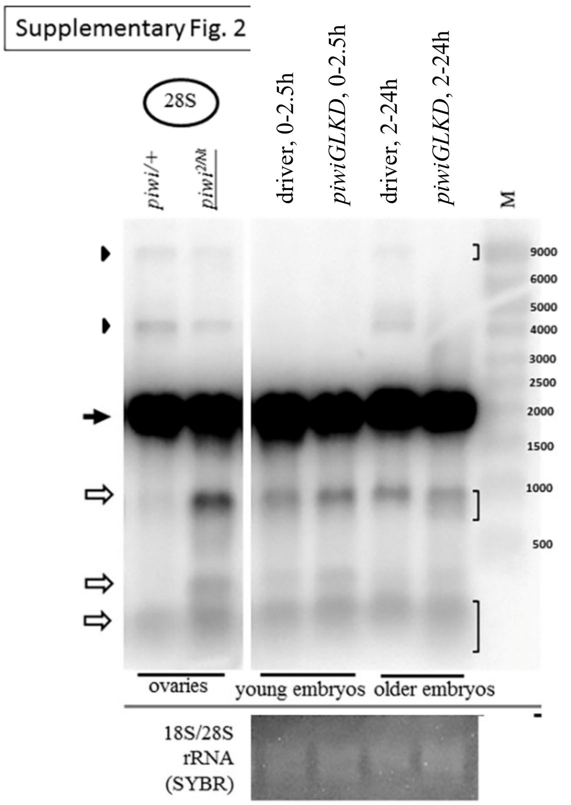

Supplementary Figure S2. rRNA fragment accumulation in *piwi* mutants is not a manifestation of the death of embryos with defects in the piRNA silencing pathway.

A probe to detect 28S rRNA 3'-end was used. In the young group 0-2.5 hours old (middle), rRNA fragments are similarly observed in control embryos of the *nos-gal4* driver fly line (labelled driver) and in embryos laid by *nos-gal4*-driven germline-specific *piwi* knockdown (*piwiGLKD*) mothers. The pre-rRNA transcript is normally not observed in young embryos because they have not initiated zygotic transcription. In the old group 2-24 hours old (right), the death of the knockdown embryos is not accompanied by the accumulation of fragments and primarily manifests itself in the absence of synthesis of the pre-rRNA (top bracket on the right), as well as in the smearing of the fragments (two lower brackets on the right).

Supplementary Fig. 3

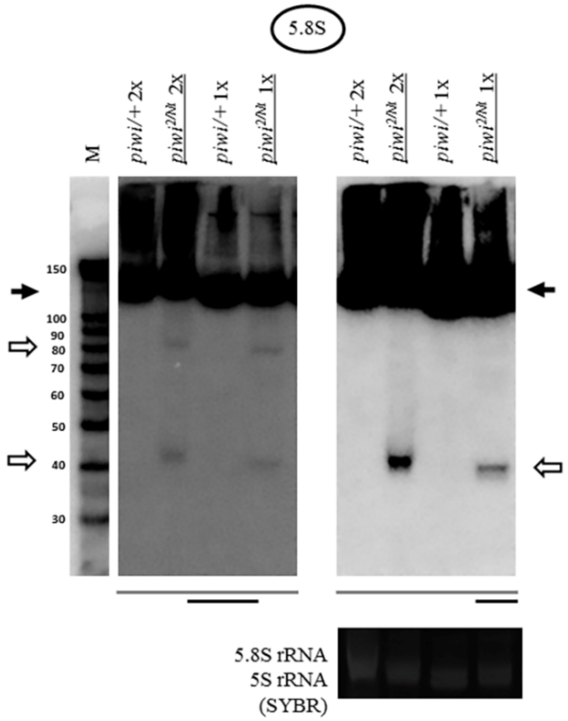

Supplementary Figure S3. Fragments from different regions of 5.8S rRNA accumulated in *piwi* mutants are different in size.

Hybridization of one blot with each of two probes detecting the middle and 3'-terminus of 5.8S rRNA 123 n. in length was carried out by sequential reprobing. On the left, a probe to the middle portion of the molecule detects two fragments ~42 n. and ~ 81 n., the sum of sizes of which equals the length of the complete molecule. On the right, a probe to the 3'-end of the molecule detects the smaller of the two fragments. Assuming the smaller of the two fragments ~42 n. long enough to be detected by both probes (by 27 n. of the 3'-end probe and by overlapping 13 n. with the middle probe), in *piwi* mutants the 5.8S rRNA molecule can be hypothesized to be split into two parts.

Supplementary Fig. 4

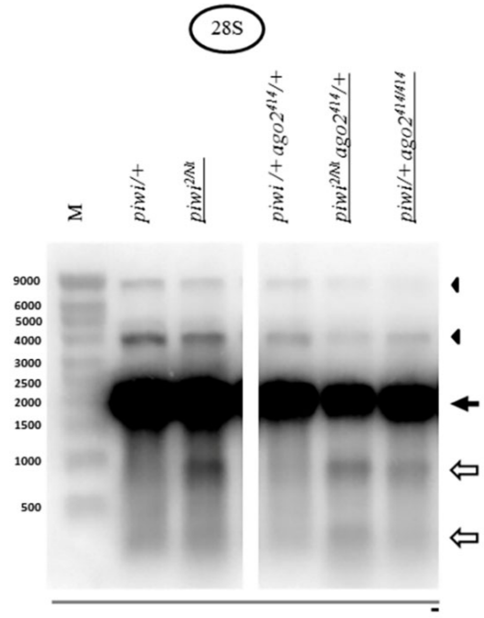

Supplementary Figure S4. Combining *piwi* and *ago2* mutations leads to rRNA fragment accumulation in *Drosophila melanogaster* ovaries.

The effect of fragment accumulation (white arrows) is observed in both *piwi*$^{2/Nt}$ mutants heterozygous for the *ago2*^{414} mutation and, to a lesser extent, in *ago2*^{414} mutants heterozygous for the *piwi* mutation. The probe used detects the 3'-end of 28S rRNA. The same amount of total RNA was loaded on the lanes of the mutants and their corresponding controls to be compared.
